# Modified life’s essential 8 mediate the correlation between dietary index for gut microbiota and sleep disorders

**DOI:** 10.3389/fnut.2025.1611714

**Published:** 2025-06-25

**Authors:** Juanjuan Fang, Zhenhua Wang, Jiangshui Yu, Yumin Ye, Apang Du, Markus W. Ferrari

**Affiliations:** ^1^The Second Affiliated Hospital of Fujian Medical University, Quanzhou, China; ^2^Department of Internal Medicine 1, Helios-HSK Clinics, Wiesbaden, Germany

**Keywords:** dietary index for gut microbiota, sleep disorders, gut microbiota, LE8, NHANES

## Abstract

**Background:**

The research sought to examine the correlation between the dietary index for gut microbiota (DI-GM) and sleep disorders. The specific relationship between DI-GM and sleep pathophysiology requires further elucidation. Methods: The data were obtained from the National Health and Nutrition Examination Survey (NHANES) across six cycles over the years 2007 to 2018. We applied logistic regression analyses with multivariable adjustments with sample weighting to assess the independent associations of DI-GM, its beneficial and unfavorable subcomponents, with sleep disorders prevalence, reporting effect estimates as adjusted odds ratios (ORs). We used restricted cubic spline (RCS) models to evaluate relationships between dose and response, and subgroup analyses to explore effect modifications. We used mediation analysis to assess the intermediary role of mLE8 (Modified Life’s Essential 8) and all its components.

**Results:**

After full adjustment, higher DI-GM and DI-GM beneficial scores were found to be associated with a reduction in sleep disorders (OR: 0.92, 95% CI: 0.86–0.97; OR: 0.90, 95% CI: 0.85–0.96, respectively). Individuals whose DI-GM was 5 or above had a 34% decreased chance of sleep disorders compared to those with scores of 3 or less (OR: 0.66, 95% CI: 0.52–0.85), while DI-GM beneficial scores ≥6 reduced the risk by 35% compared to those with scores ≤1 (OR: 0.65, 95% CI: 0.49–0.85). RCS showed a non-linear negative trend for DI-GM (*p* < 0.001; nonlinear *p* = 0.046) and a non-linear association for DI-GM beneficial (*p* < 0.001; non-linear *p* = 0.023) with sleep disorders. Subgroup analyses confirmed the robustness of these associations among male individuals, current smokers, individuals consuming ≥12 alcoholic drinks/year, and those without hypertension, diabetes, or depression (*p* < 0.05). After full adjustment for covariates, mLE8 exhibited a significant mediating role in the associations of both DI-GM (14.46% mediated effect, *p* < 0.001) and DI-GM beneficial (12.99% mediated effect, *p* < 0.001) with sleep disorders. With all components of mLE8, only the nicotine exposure mediated 3.44% of the association between DI-GM and sleep disorders.

**Conclusion:**

Elevated DI-GM and DI-GM beneficial scores are associated with reduced incidence of sleep disorders. mLE8 mediates the associations of both DI-GM and DI-GM beneficial with sleep disorders.

## 1 Introduction

Sleep disorders have become a significant public health problem globally, with epidemiological data highlighting their widespread prevalence. Studies indicate that approximately 33% of the global population experiences insomnia, while between 10 and 15% of individuals experience chronic insomnia. These results highlight the pressing necessity for action and additional studies to tackle this escalating public health issue (1, 2). In specific disease populations (e.g., Parkinson’s disease, diabetes, and Tourette syndrome), the prevalence of sleep disorders can reach 71–77.5% (3–5). Neuropsychiatric disorders, including stroke, depression, and anxiety, along with neurodegenerative diseases like Alzheimer’s, and metabolic conditions such as diabetes, frequently accompany sleep disorders, leading to a detrimental cycle. For instance, the incidence of sleep disorders is markedly elevated in individuals with depression and may predict disease progression (6–8). The impact of sleep disorders spans physiological health, mental well-being, quality of life, and socioeconomic burden. Physiologically, sleep disorders elevate cardiovascular and metabolic risks. Studies demonstrate that sleep disorders are linked to sympathetic overactivation, increasing risks of hypertension, arteriosclerosis, and cardiovascular events (9). Among obstructive sleep apnea (OSA) patients, 60% exhibit worsened diabetes control, and sleep disorders directly exacerbate the progression of diabetes and its complications (10, 11). Sleep disorders also impair neurological health; OSA is associated with heightened stroke risk (12), while chronic sleep deprivation (SD) may contribute to cognitive impairment, white matter impairments, and Alzheimer’s disease (7). Furthermore, chronic sleep disorders weaken immune function and may elevate cancer risk (13). Psychologically, sleep disorders significantly increase psychotic experiences, depression, anxiety, and fatigue, particularly among adolescents, where sleep issues and smartphone addiction synergistically worsen mental health (14). Additionally, sleep disorders severely impair quality of life and amplify socioeconomic burdens, with over 50% of patients experiencing comorbid conditions (e.g., insomnia coupled with circadian rhythm disorders), leading to daytime drowsiness, reduced productivity, and diminished social engagement (15).

Longitudinal cohort studies reveal dynamic interactions between sleep, brain, and gut microbiota beginning in infancy. A study tracking 162 healthy infants showed that daytime sleep duration significantly correlates with gut microbiota *α*-diversity (*p* < 0.05), while nighttime sleep continuity associates with specific microbial taxa abundance (16), suggesting co-evolution of sleep patterns and gut microbiota early in life. In adults, large-scale population-based research (*n* = 1,809) identified altered chronic insomnia patients’ abundances of *Ruminococcaceae UCG-002/UCG-003,* with dose–response relationships to bile acid metabolism dysregulation (17). Such microbiota–metabolite axis disruptions may mediate links between sleep disorders and cardiometabolic diseases.

As a complex ecosystem, the gut microbiota is affected by the host’s genetic makeup, environmental factors, and dietary habits, with dietary patterns exerting a dominant influence on microbial metabolic activity and homeostasis (18, 19). Traditional approaches, such as the sulfur microbial diet, which tracks sulfate-reducing bacterial pathways, highlight links between dietary sulfur and specific microbiota–metabolite interactions (20, 21). However, such targeted methods fail to capture the holistic impact of diet on microbial ecology. This new indicator of dietary index for gut microbiota (DI-GM) addresses this gap by integrating multidimensional parameters to quantify dietary quality and microbiota health (22). High DI-GM scores correlate with enhanced gut microbiota *α*-diversity, increased SCFA-producing taxa (e.g., Ruminococcus and Prevotella), and reduced pro-inflammatory bacteria (e.g., Desulfovibrionaceae) (19, 23, 24). Unlike single-dimensional indices, DI-GM dynamically reflects functional markers like SCFA synthesis and the Firmicutes/Bacteroidetes ratio, offering a comprehensive tool to study diet–microbiota interactions in long-term conditions like metabolic syndrome and heart diseases (18, 23, 25).

The American Heart Association (AHA) has crafted Life’s Essential 8 (LE8) as a comprehensive approach to optimize heart health and encourage longevity through lifestyle adjustments. LE8 evaluates eight key health metrics: quality of diet, levels of physical activity, nicotine intake, sleep routines, weight management, lipid profiles, glucose concentrations, and blood pressure management (26). By addressing these interconnected dimensions, LE8 provides a structured approach to improving overall health and extending the duration of life free from chronic disease. Recent studies suggest that the DI-GM may influence sleep quality via multiple LE8 dimensions. For instance, DI-GM improves insulin sensitivity and lipid metabolism, thereby reducing the risks of obesity and metabolic syndrome, which indirectly mitigates sleep disorders (27, 28).

Nevertheless, evidence linking DI-GM to sleep disorders remains scarce. Existing studies suggest that gut microbiota influences sleep rhythms through metabolite secretion and neuroimmune pathways (29, 30). However, population-level analyses quantifying the DI-GM in relation to sleep disorders risks remain scarce. Notably, associations between prefrontal cortex gray matter thickness and slow-wave sleep highlight how the microbiota–gut–brain axis (MGBA) contributes to sleep regulation (30). To address these gaps, this investigation uses data sourced from the American National Health and Nutrition Examination Survey (NHANES) to explore the link between DI-GM scores and the risk of sleep disorders, offering fresh insights into how dietary patterns that influence gut microbiota may contribute to sleep disorders. Furthermore, this study will examine the probable mediation of LE8 in this relationship.

## 2 Materials and methods

### 2.1 Data source

Conducted by the National Center for Health Statistics, the NHANES provides a representative evaluation of the U. S. population, delivering both cross-sectional and longitudinal insights into health and nutrition. From 2007 through 2018, 59,842 individuals were enrolled by NHANES. For this research, certain participants were left out if they lacked assessment data regarding sleep disorders (*n* = 28,242), had missing values for DI-GM, DI-GM beneficial, DI-GM unfavorable, and mLE8 (*n* = 7,684), or possessed incomplete covariate data (*n* = 9,241). Following a careful application of the conditions for exclusion, for analysis, the sample was composed of 14,675 individuals (Supplementary Figure 1).

### 2.2 Sleep disorders

To assess the sleep disorders, it was conducted using self-reported responses to the inquiry: ‘During the last 2 weeks, how often have you experienced problems like trouble falling asleep, staying asleep, or sleeping excessively?’ Those who mentioned experiencing these issues ‘more than half the days’ or ‘nearly every day’ were identified as having sleep disorders. People who replied with ‘several days’ or ‘not at all’ were identified as not suffering from sleep disorders (31). To minimize bias as much as possible, we further included objective sleep-related measures (sleep duration) as covariates in the study.

### 2.3 DI-GM

We used the two 24-h dietary recalls to determine the DI-GM. DI-GM encompasses 14 dietary components, including beneficial items such as fermented dairy products, chickpeas, soybeans, whole grains, dietary fiber, cranberries, avocados, broccoli, coffee, and green tea, as well as unfavorable items such as refined grains, red meat, processed meats, and high-fat diets. A high-fat diet was defined as one where fat contributes ≥40% of total energy intake. As specific tea consumption data were unavailable in NHANES dietary recalls, the DI-GM scoring system, with a maximum possible score of 14, spans from 0 up to 13. Detailed scoring criteria and calculation methods are provided in Supplementary Table 1 (22).

### 2.4 mLE8

Developed by the AHA, LE8 operationalizes cardiovascular health through eight key health metrics: quality of diet, levels of physical activity, nicotine intake, sleep routines, weight management, lipid profiles, glucose concentrations, and blood pressure management. The standardized scoring protocol for LE8 components, derived from NHANES data using AHA-defined criteria, is detailed in Supplementary Table 2. Specifically, each component is scored 0–100 based on established clinical thresholds. The composite LE8 score is determined by averaging all eight sub-scores equally. The dietary composition and assessment metrics in DI-GM differ from those in LE8, so we retained the dietary component of LE8. Since sleep in LE8 is directly related to the outcome of sleep disorders in this study, we excluded sleep from LE8 and defined a new variable, modified LE8.

The dietary component was evaluated using the Index for Healthy Eating in 2015 (HEI-2015) (32), with its constituent dietary elements and scoring criteria comprehensively outlined in Supplementary Table 3. HEI-2015 scores were derived through standardized analysis of dietary recall data for 24 h collected during mobile examination center visits. Concurrently, self-administered questionnaires captured behavioral metrics (physical activity patterns, nicotine exposure status, sleep quality parameters) and clinical histories (diabetes diagnosis and medication use). During standardized physical examinations, trained staff obtained triplicate blood pressure measurements using validated protocols, while anthropometric data were professionally recorded to determine body mass index (BMI). Fasting venous blood samples were subsequently analyzed in certified laboratories for lipid profiles and glucose homeostasis markers (plasma glucose and glycated hemoglobin), ensuring biochemical parameter quantification met NIH quality assurance standards.

### 2.5 Covariates

Covariates encompassed demographic factors (age, sex, poverty-income ratio, race, education, and marital status), ways of living [smoking, alcohol use, BMI, and metabolic equivalent of task (MET)] categorization (33), and clinical conditions (hypertension, diabetes, depression, and stroke). Hypertension was identified in participants who met one or more of the following criteria: average systolic/diastolic blood pressure ≥140/90 mmHg, physician-confirmed diagnosis, or antihypertensive medication use (34, 35). Diabetes was identified by fasting glucose levels of 7 mmol/L or more, random glucose levels of 11.1 mmol/L or more, 2-h OGTT levels of 11.1 mmol/L or more, HbA1c levels of 6.5% or more, a clinical diagnosis, or hypoglycemic therapy (36, 37). The severity of depression was evaluated with the 9-item Patient Health Questionnaire (PHQ-9), with clinically significant symptoms defined as scores ≥10 [88% sensitivity/specificity for major depressive disorder (38)]. Stroke history was self-reported via the Medical Condition Questionnaire (MCQ) (39).

### 2.6 Statistical analysis

The data were processed and analyzed using R (version 4.5.0) and Zstats v0.90.^1^ To ensure national representativeness, we followed the National Center for Health Statistics (NCHS) recommendations for sampling weights, specifically utilizing the sample weight of the two-day dietary (WTDR 2D). For pooled analyses across NHANES cycles (2007–2018), a weighting adjustment factor of 1/6 × WTDR 2D was implemented in accordance with NCHS guidelines. Weighted means ± standard errors (SEs) were used to express continuous variables, whereas variables that fall into distinct categories were presented as frequencies. To evaluate the associations between exposure variables (DI-GM, DI-GM beneficial, DI-GM unfavorable, and mLE8) and sleep disorders, multivariable models utilizing logistic regression were utilized. The findings were reported in terms of adjusted odds ratios (ORs) and 95% confidence intervals. For the sake of consistency in the connection, we sorted DI-GM and DI-GM beneficial into four groups, whereas DI-GM unfavorable and mLE8 were categorized into three groups based on tertiles. Trend tests were conducted, and corresponding *p*-values were computed.

To address the research objectives, we employed a structured analytical approach involving three multivariable logistic regression models, each incrementally adjusted for potential confounders. Model 1 was an unadjusted baseline model. Model 2 included adjustments for demographic variables such as age, gender, race, marital status, education level, and poverty–income ratio (PIR). Model 3, the fully adjusted model, further accounted for lifestyle factors (smoking status and alcohol consumption in the previous year), anthropometric measures (BMI), physical activity (MET categorization), sleep duration, and chronic conditions (hypertension, diabetes, depression, and stroke). Restricted cubic splines (RCSs) were used to investigate possible non-linear relationships.

In subgroup analyses, we maintained consistency by adjusting for the same set of covariates: gender, age, education level, race, PIR, marital status, smoking status, alcohol use, BMI, MET categorization, sleep duration, hypertension, depression, diabetes, and stroke. This approach ensured the robustness of our findings across different population strata.

Finally, to evaluate the intermediary role of mLE8 in the connections within DI-GM, DI-GM beneficial, and sleep disorders, we employed Bayesian resampling methodology. We used the ‘mediation’ package in R to perform the mediation analysis, with 1,000 Bayesian resamples to estimate effects. The following formula was used to calculate the mediation effect: (Indirect effect / (Indirect effect + Direct effect)) × 100% (40).

## 3 Results

### 3.1 Baseline characteristics

The final analysis included 14,675 participants, with 2,357 classified as having sleep disorders. Baseline characteristics stratified by sleep disorders status revealed significantly lower DI-GM and DI-GM beneficial scores in the sleep disorders (*p* < 0.001). Substantial distinctions between the groups (*p* < 0.05) were observed in sex, poverty-income ratio (PIR), race, education, marital status, smoking, BMI, MET categories, sleep duration, and prevalence of hypertension, diabetes, depression, and stroke. No statistically significant differences emerged in age, alcohol consumption, or DI-GM Unfavorable scores (*p* > 0.05). Furthermore, for mLE8 and all its components, excluding blood lipids, scores were significantly lower in the sleep disorders group (*p* < 0.05) (Table 1).

**Table 1:** Population features classified in accordance with sleep disorders.

Variable	Total (*n* = 14,675)	Non-sleep disorders (*n* = 12,318)	Sleep disorders (*n* = 2,357)	Statistic	*P*
Age, mean (SE), years	47.13 (0.34)	47.05 (0.38)	47.59 (0.50)	t = 0.96	0.343
Gender, *n* (%)				χ^2^ = 64.45	<0.001
Male	7,084 (48.06)	6,129 (49.48)	955 (40.33)		
Female	7,591 (51.94)	6,189 (50.52)	1,402 (59.67)		
Marital status, *n* (%)				χ^2^ = 121.77	<0.001
Married	7,803 (56.26)	6,774 (57.85)	1,029 (47.63)		
Widowed	1,118 (5.14)	916 (4.86)	202 (6.63)		
Divorced	1,613 (10.61)	1,239 (9.90)	374 (14.50)		
Separated	453 (2.00)	344 (1.72)	109 (3.55)		
Never married	2,623 (18.89)	2,171 (18.74)	452 (19.72)		
Living with partner	1,065 (7.10)	874 (6.94)	191 (7.96)		
PIR, mean (SE)	3.02 (0.05)	3.11 (0.05)	2.53 (0.07)	t = −9.19	<0.001
Race, *n* (%)				χ^2^ = 26.78	<0.001
Mexican American	2040 (8.19)	1778 (8.48)	262 (6.59)		
Other Hispanic	1,367 (5.07)	1,113 (4.83)	254 (6.32)		
Non-Hispanic White	7,050 (69.86)	5,834 (69.80)	1,216 (70.19)		
Non-Hispanic Black	2,944 (10.45)	2,463 (10.23)	481 (11.68)		
Other Race—Including Multi-Racial	1,274 (6.43)	1,130 (6.66)	144 (5.22)		
Education level, *n* (%)				χ^2^ = 139.27	<0.001
Less than high school	3,339 (15.33)	2,641 (14.12)	698 (21.94)		
High school or equivalent	3,326 (22.13)	2,735 (21.45)	591 (25.85)		
College or above	8,010 (62.54)	6,942 (64.43)	1,068 (52.21)		
BMI, mean (SE), kg·m^−2^	28.92 (0.10)	28.72 (0.11)	30.03 (0.21)	t = 6.19	<0.001
Smoking history, *n* (%)				χ^2^ = 159.44	<0.001
No	8,265 (57.16)	7,189 (59.36)	1,076 (45.11)		
Yes	6,410 (42.84)	5,129 (40.64)	1,281 (54.89)		
Drank at least 12 alcoholic drinks in the previous year, *n* (%)				χ^2^ = 0.38	0.631
No	3,991 (22.20)	3,369 (22.11)	622 (22.69)		
Yes	10,684 (77.80)	8,949 (77.89)	1735 (77.31)		
MET categorization, *n* (%)				χ^2^ = 106.55	<0.001
Low physical activity	5,642 (34.13)	4,535 (32.40)	1,107 (43.57)		
High physical activity	9,033 (65.87)	7,783 (67.60)	1,250 (56.43)		
Sleep duration, mean (SE), hours	6.90 (0.02)	7.02 (0.02)	6.27 (0.07)	t = −10.46	<0.001
Hypertension, *n* (%)				χ^2^ = 56.97	<0.001
No	8,450 (63.00)	7,266 (64.28)	1,184 (55.97)		
Yes	6,225 (37.00)	5,052 (35.72)	1,173 (44.03)		
Diabetes, *n* (%)				χ^2^ = 36.38	<0.001
No	11,970 (86.86)	10,167 (87.58)	1803 (82.93)		
Yes	2,705 (13.14)	2,151 (12.42)	554 (17.07)		
Depression, *n* (%)				χ^2^ = 3702.75	<0.001
No	13,351 (91.97)	11,994 (97.81)	1,357 (60.07)		
Yes	1,324 (8.03)	324 (2.19)	1,000 (39.93)		
Stroke, *n* (%)				χ^2^ = 49.85	<0.001
No	14,158 (97.37)	11,950 (97.77)	2,208 (95.20)		
Yes	517 (2.63)	368 (2.23)	149 (4.80)		
DI-GM, score, mean (SE)	5.20 (0.03)	5.25 (0.04)	4.92 (0.06)	t = −5.17	<0.001
DI-GM Beneficial, score, mean (SE)	2.38 (0.03)	2.43 (0.03)	2.11 (0.04)	t = −7.46	<0.001
DI-GM Unfavorable, score, mean (SE)	2.61 (0.02)	2.61 (0.02)	2.62 (0.04)	t = 0.17	0.865
mLE8, score, mean (SE)	62.92 (0.29)	63.91 (0.27)	57.48 (0.47)	t = −15.33	<0.001
DI-GM group, *n* (%)				χ^2^ = 66.20	<0.001
0–3	2,574 (15.68)	2,117 (15.00)	457 (19.40)		
4	3,042 (19.43)	2,493 (18.84)	549 (22.63)		
5	3,422 (22.95)	2,878 (22.99)	544 (22.68)		
≥6	5,637 (41.94)	4,830 (43.16)	807 (35.30)		
DI-GM Beneficial group, *n* (%)				χ^2^ = 88.00	<0.001
0–1	4,682 (27.66)	3,817 (26.42)	865 (34.43)		
2–3	7,208 (51.16)	6,066 (51.37)	1,142 (50.03)		
4–5	1927 (14.67)	1,679 (15.27)	248 (11.40)		
≥6	858 (6.51)	756 (6.95)	102 (4.14)		
DI-GM Unfavorable group, *n* (%)				χ^2^ = 0.30	0.935
0–1	2,266 (15.41)	1905 (15.36)	361 (15.72)		
2	3,685 (26.01)	3,089 (25.98)	596 (26.18)		
≥ 3	8,724 (58.58)	7,324 (58.66)	1,400 (58.10)		
mLE8 group, *n* (%)				χ^2^ = 397.72	<0.001
T1	5,391 (32.34)	4,150 (29.30)	1,241 (48.90)		
T2	4,954 (34.02)	4,232 (34.41)	722 (31.87)		
T3	4,330 (33.65)	3,936 (36.29)	394 (19.23)		
Physical activity score, mean (SE)	73.31 (0.67)	75.11 (0.68)	63.47 (1.42)	t = −8.05	<0.001
Nicotine exposure score, mean (SE)	72.13 (0.73)	74.54 (0.63)	58.96 (1.74)	t = −9.53	<0.001
Sleep score, mean (SE)	82.43 (0.39)	85.38 (0.33)	66.37 (1.05)	t = −17.46	<0.001
Blood lipids score, mean (SE)	68.93 (0.45)	69.05 (0.44)	68.28 (1.16)	t = −0.68	0.500
Glucose score, mean (SE)	84.90 (0.33)	85.52 (0.31)	81.57 (0.72)	t = −5.89	<0.001
Blood pressure score, mean (SE)	87.85 (0.29)	88.15 (0.31)	86.21 (0.56)	t = −3.24	0.002
Diet score, mean (SE)	38.50 (0.60)	39.62 (0.65)	32.36 (0.76)	t = −7.71	<0.001
BMI score, mean (SE)	61.24 (0.52)	62.25 (0.55)	55.71 (1.01)	t = −6.06	<0.001

Mean (SE) is used to present continuous variables, while frequency is used for categorical variables. The DI-GM and DI-GM beneficial scores were categorized into four groups. DI-GM unfavorable score and mLE8 score were categorized into three groups. Abbreviations: BMI, body mass index; MET, metabolic equivalent of task; PIR, poverty income ratio; DI-GM, dietary index for gut microbiota; mLE8: Modified Life’s Essential 8; SE, Standard Error; t: *t*-test, χ^2^: Chi-square test.

### 3.2 The associations between DI-GM, DI-GM beneficial, DI-GM unfavorable, and mLE8 with sleep disorders

Significant inverse links between the DI-GM and sleep disorders were found through multivariable logistic regression analyses across progressively adjusted models: unadjusted (Model 1: OR = 0.89, 95% CI: 0.86–0.93), adjusted for sociodemographic factors (Model 2: OR = 0.92, 95% CI: 0.87–0.96), and fully adjusted for factors associated with lifestyle and clinical conditions (Model 3: OR = 0.92, 95% CI: 0.86–0.97). Our findings indicate that each additional unit of DI-GM is linked to an 8% decline in the prevalence of sleep disorders. When arranged into four sections according to DI-GM scores, fully adjusted models demonstrated that individuals with DI-GM scores of 5 or higher experienced at least a 34% lower risk of developing sleep disorders than those with scores of 3 or less (OR: 0.66, 95% CI: 0.52–0.85; *P* for trend = 0.004). Similarly, DI-GM beneficial showed consistent inverse relationships in all models (Model 1: OR = 0.84, 95% CI: 0.80–0.88; Model 2: OR = 0.88, 95% CI: 0.84–0.93; Model 3: OR = 0.90, 95% CI: 0.85–0.96), with group analysis revealing a 35% risk reduction for scores ≥6 compared to those with scores ≤1 (OR: 0.65, 95% CI: 0.49–0.85; *P* for trend = 0.021). In contrast, no significant associations were observed between DI-GM unfavorable and sleep disorders in any model or stratified assessment (*p* > 0.05). The results demonstrated a significant inverse connection between mLE8 scores and sleep disorders, with an odds ratio of 0.98 (95% CI: 0.97–0.99). Specifically, participants with the top mLE8 scores exhibited an average prevalence of sleep disorders that was 0.38 units lower when compared with the group that had the lowest scores (OR = 0.62, 95% CI: 0.47–0.81). Moreover, a trend test in the model verified the link between mLE8 scores and the frequency of sleep disorders in relation to dosage, achieving statistical significance (*p* < 0.05) (Table 2).

**Table 2:** The associations between DI-GM, DI-GM beneficial, DI-GM unfavorable, and mLE8 with sleep disorders.

Variables	Model 1		Model 2		Model 3	
	OR (95%CI)	*P*	OR (95%CI)	*P*	OR (95%CI)	*P*
DI-GM score	0.89 (0.86 ~ 0.93)	<0.001	0.92 (0.87 ~ 0.96)	<0.001	0.92 (0.86 ~ 0.97)	0.005
DI-GM group						
0–3	1.00 (Reference)		1.00 (Reference)		1.00 (Reference)	
4	0.93 (0.74 ~ 1.17)	0.537	0.93 (0.74 ~ 1.18)	0.576	0.83 (0.62 ~ 1.11)	0.211
5	0.76 (0.61 ~ 0.95)	0.019	0.80 (0.64 ~ 1.01)	0.070	0.66 (0.52 ~ 0.85)	0.002
≥6	0.63 (0.51 ~ 0.79)	<0.001	0.70 (0.56 ~ 0.89)	0.004	0.65 (0.50 ~ 0.86)	0.005
Trend test		<0.001		<0.001		0.004
DI-GM beneficial score	0.84 (0.80 ~ 0.88)	<0.001	0.88 (0.84 ~ 0.93)	<0.001	0.90 (0.85 ~ 0.96)	0.001
DI-GM beneficial group						
0–1	1.00 (Reference)		1.00 (Reference)		1.00 (Reference)	
2–3	0.75 (0.66 ~ 0.84)	<0.001	0.84 (0.74 ~ 0.95)	0.008	0.81 (0.69 ~ 0.95)	0.013
4–5	0.57 (0.46 ~ 0.72)	<0.001	0.67 (0.53 ~ 0.86)	0.002	0.77 (0.57 ~ 1.04)	0.095
≥6	0.46 (0.35 ~ 0.60)	<0.001	0.56 (0.42 ~ 0.75)	<0.001	0.65 (0.49 ~ 0.85)	0.003
Trend test		<0.001		<0.001		0.021
DI-GM unfavorable score	1.01 (0.93 ~ 1.09)	0.865	0.99 (0.91 ~ 1.07)	0.819	0.97 (0.89 ~ 1.06)	0.495
DI-GM unfavorable group						
0–1	1.00 (Reference)		1.00 (Reference)		1.00 (Reference)	
2	0.98 (0.81 ~ 1.19)	0.870	0.98 (0.81 ~ 1.20)	0.870	1.03 (0.83 ~ 1.27)	0.787
≥ 3	0.97 (0.80 ~ 1.17)	0.732	0.94 (0.77 ~ 1.14)	0.523	0.93 (0.74 ~ 1.17)	0.531
mLE8 score	0.96 (0.96 ~ 0.97)	<0.001	0.97 (0.96 ~ 0.97)	<0.001	0.98 (0.97 ~ 0.99)	0.002
mLE8 group						
T1	1.00 (Reference)		1.00 (Reference)		1.00 (Reference)	
T2	0.56 (0.47 ~ 0.65)	<0.001	0.61 (0.53 ~ 0.70)	<0.001	0.83 (0.68 ~ 1.01)	0.067
T3	0.32 (0.27 ~ 0.38)	<0.001	0.36 (0.31 ~ 0.42)	<0.001	0.62 (0.47 ~ 0.81)	0.001
Trend test		<0.001		<0.001		<0.001

Model 1: Crude; Model 2: Adjusted: age, marital status, gender, race, education level, PIR; Model3: Adjusted: age, PIR, gender, race, marital status, education level, smoking status, drank at least 12 alcoholic drinks in the previous year, BMI, MET categorization, sleep duration, hypertension, diabetes, depression, stroke; Abbreviations: BMI, body mass index; MET, metabolic equivalent of task; PIR, poverty income ratio; DI-GM, dietary index for gut microbiota; mLE8, Modified Life’s Essential 8; OR, odds ratio; CI, Confidence Interval.

### 3.3 The dose–response associations between DI-GM, DI-GM beneficial, DI-GM unfavorable, and mLE8 with sleep disorders

To examine the dose–response connections between DI-GM, DI-GM beneficial, DI-GM unfavorable, and mLE8 with sleep disorders, we employed RCSs while considering all covariates. According to the RCS analysis, a notable inverse non-linear correlation exists between DI-GM scores and the prevalence of sleep disorders (*p* < 0.001, *P* for non-linear = 0.046). The DI-GM beneficial exhibits a significant non-linear negative association with sleep disorders (*p* < 0.001, *P* for non-linear = 0.023). Additionally, sleep disorders are significantly negatively associated with the mLE8 in a linear manner (*p* < 0.001, *P* for non-linear = 0.123). Our analysis did not find a statistically significant connection between DI-GM unfavorable scores and sleep disorders (Supplementary Figure 3).

### 3.4 Sensitivity and subgroup analysis

The research investigates the persistence of the relationship between DI-GM and DI-GM beneficial with sleep disorders, in addition to possible interactions. Sensitivity and subgroup analyses stratified by sex, smoking status, alcohol use, and clinical comorbidities (hypertension, diabetes, depression, and stroke) were executed to analyze the links of DI-GM and DI-GM beneficial with sleep disorders. All models were modified to account for covariates, excluding the factors used for stratification. Subgroup analyses revealed that both DI-GM and DI-GM beneficial showed statistically significant negative associations with sleep disorders among males, current smokers, individuals consuming ≥12 alcoholic drinks/year, and those without hypertension, diabetes, or depression (*p* < 0.05). Regardless of stroke status, DI-GM and DI-GM beneficial were significantly negatively associated with sleep disorders. Additionally, the relationship between DI-GM and sleep disorders revealed a significant interaction with smoking (*P* for interaction = 0.045). Meanwhile, the relationship between DI-GM beneficial and sleep disorders revealed a significant interaction with smoking and drinking (*P* for interaction < 0.05).

### 3.5 Mediation effect analysis

A significant relationship was found between DI-GM, DI-GM beneficial, and mLE8 after adjusting for all covariates (*β* = 1.13, 95% CI: 0.99–1.28, *p* < 0.001; *β* = 1.15, 95% CI: 1.99–1.30, *p* < 0.001, respectively) (Table 3). According to the assumptions, we found that mLE8 plays a mediating role after adjusting for covariates (Supplementary Figure 4). mLE8 represented 14.46% of the link between DI-GM and sleep disorders. Similarly, 12.99% of the connection between DI-GM beneficial and sleep disorders was mediated by mLE8 (*p* < 0.001). Additionally, we further examined the mediating effects of individual mLE8 components on the relationships between DI-GM, DI-GM beneficial, and sleep disorders. Statistical analyses revealed that only the nicotine exposure mediated 3.44% of the association between DI-GM and sleep disorders (*p* < 0.05), with no significant mediation effects observed for other components.

**Table 3:** A regression model that uses several variables on DI-GM, DI-GM beneficial, and mLE8.

Variables	β (95%CI)	*P*
DI-GM - mLE8	1.13 (0.99 ~ 1.28)	<0.001
DI-GM beneficial - mLE8	1.15 (1.00 ~ 1.30)	<0.001

Analyses were adjusted for gender, age, race, marital status, PIR, education level, smoking status, drank at least 12 alcoholic drinks in the previous year, BMI, MET, and sleep duration. Categorization, hypertension, diabetes, depression, and stroke. mLE8: Modified Life’s Essential 8; DI-GM, dietary index for gut microbiota; CI: Confidence Interval.

## 4 Discussion

The study investigated the connection between DI-GM and sleep disorders, based on NHANES data ranging from 2007 to 2018. These results indicated a robust negative connection between DI-GM and DI-GM beneficial scores and the risk of developing sleep disorders. Specifically, those with DI-GM scores of 5 or above showed a 34% or greater reduction in sleep disorders compared to those scoring 3 or below. Similarly, people with DI-GM beneficial scores of 6 or higher had a 35% lower risk than those with scores of 1 or less. Conversely, the analyses did not reveal any significant correlations between DI-GM unfavorable and sleep disorders. Subgroup analyses revealed that both DI-GM and DI-GM beneficial showed statistically significant negative associations with sleep disorders among males, current smokers, individuals consuming ≥12 alcoholic drinks/year, and those without hypertension, diabetes, or depression. The relationship between DI-GM and sleep disorders revealed a significant interaction with smoking. Meanwhile, the relationship between DI-GM beneficial and sleep disorders revealed a significant interaction with smoking and drinking. According to the findings, mLE8 appears to play a mediating role in the link between DI-GM, DI-GM beneficial, and sleep disorders. Elevated DI-GM scores correspond to a diminished prevalence of sleep disorders, with mLE8 playing a pivotal role in bridging this connection. These results affirm our initial hypothesis and underscore the significance of mLE8 as a mediating element in the connection between nutritional indices and sleep well-being.

The investigation focuses on the correlation between DI-GM and sleep disorders. Our results align with prior investigations linking gut microbiota diversity to sleep disturbances. To begin with, clinical studies consistently show that gut dysbiosis is present in patients suffering from insomnia and OSA, and other sleep disorders. For example, insomnia patients exhibit reduced abundances of Ruminococcaceae UCG-002 and UCG-003, which relate to the dysregulation of bile acid metabolism and may mediate cardiovascular metabolic risks through the microbiota–bile acid axis (17). OSA patients demonstrate depleted butyrate-producing bacteria (e.g., Roseburia and Faecalibacterium), with hypoxia severity (rather than apnea frequency) driving species-specific microbiota alterations (41, 42). Patients with type 1 narcolepsy (NT1) exhibited a significant difference in global bacterial community structure compared to controls (43). Second, microbiota diversity may predict sleep quality. A study in older adults found gut microbiota *α*-diversity (e.g., Shannon index) positively correlated with actigraphy-measured sleep quality, independent of age and lifestyle factors (44). Animal studies further support microbiota–sleep interactions. Comparisons between specific pathogen-free (SPF) and germ-free (GF) mice revealed that microbiota depletion alters sleep architecture. SD reduced fecal and hypothalamic butyrate levels in SPF mice but not GF mice, suggesting butyrate mediates sleep regulation via the MGBA (45). Chronic SD induces microbiota dysbiosis, activates NLRP3 inflammasomes found in the brain and colon, and impairs the function of the intestinal and blood–brain barriers, ultimately impairing cognition. Fecal microbiota transplantation confirmed that SD-induced dysbiosis directly drives cognitive deficits, highlighting the MGBA in sleep-related neurodegeneration (46, 47). SD-associated dysbiosis also promotes neuroinflammation (e.g., microglial activation AND elevated IL-1β/TNF-*α*), partially reversible via probiotic supplementation (48).

Current studies reveal that the association between gut microbiota and sleep disorders involves sophisticated two-way regulatory mechanisms. The DI-GM score primarily serves as an index of the variety of gut microbiota and is strongly linked to the synthesis of SCFAs. This interaction is likely mediated by the MGBA, encompassing multiple pathways such as metabolic, immune, and neural signaling. First, metabolic pathways: gut microbiota-derived SCFAs (e.g., butyrate and propionate) penetrate the blood–brain barrier to modulate the function of the central nervous system. Butyrate alleviates hypothalamic metabolic disturbances induced by SD and improves sleep architecture via vagus nerve activation (45, 49). Chronic insomnia correlates with bile acid dysregulation, where specific taxa (e.g., Ruminococcaceae) influence cardiometabolic risks through bile acid metabolism, underscoring the metabolic axis in sleep disorders (17). Additionally, microbiota metabolites (e.g., tryptophan and serotonin precursors) regulate serotonin and melatonin synthesis, directly modulating sleep–wake cycles (50, 51). Second, immune and inflammatory pathways: SD compromises intestinal barrier integrity (e.g., reduced tight junction proteins), enabling bacterial byproducts (e.g., lipopolysaccharides) to trigger systemic inflammation (e.g., NF-κB activation) and impair sleep quality (46–48). Chronic sleep restriction has been shown to activate NLRP3 inflammasomes in the colon and brain, resulting in the secretion of pro-inflammatory cytokines, including IL-1β and IL-18. This inflammatory cascade contributes to neuroinflammation and is associated with cognitive impairments (46, 47, 49). Gut microbiota also modulates Th17/Treg balance and IL-17 secretion, indirectly influencing immune status in sleep-related brain regions (e.g., hypothalamus) (47, 49). Additionally, neural pathways: microbiota-derived signals are transmitted to the central nervous system via the vagus nerve, regulating hypothalamic–pituitary–adrenal (HPA) axis activity, cortisol levels, and stress responses, thereby disrupting circadian rhythms (45, 50, 52). Microbial metabolites (e.g., *γ*-aminobutyric acid, GABA) directly stimulate enterochromaffin cells to synthesize serotonin and modulate sleep-associated neurotransmitters (e.g., dopamine and norepinephrine) through the gut–brain axis (50, 51).

As a novel cardiovascular health metric, the relationship between LE8 and gut microbiota/sleep disorders remains underexplored. This investigation may provide significant evidence regarding the intersection of the “gut–brain axis” with cardiovascular health (53, 54). Current research suggests LE8 exhibits reciprocal interactions with sleep health across multiple dimensions. First, sleep itself—as an independent LE8 component—directly modulates cardiovascular risk via quantitative metrics (e.g., recommended 7–9 h duration) and qualitative metrics (e.g., reduced sleep fragmentation). SD lowers LE8 scores and activates inflammatory pathways (55–57). Second, metabolic indicators (BMI, blood glucose, and non-HDL cholesterol) exhibit bidirectional relationships with sleep. Obesity disrupts sleep homeostasis through chronic inflammation, while sleep insufficiency exacerbates insulin resistance and lipid dysregulation. Concurrently, HPA axis dysregulation (e.g., abnormal cortisol rhythms) further impairs sleep (58). Third, the behavioral components of LE8, including physical activity, dietary patterns, and smoking cessation, act synergistically to improve sleep architecture through distinct yet complementary biological mechanisms: regular exercise enhances adenosine accumulation that promotes sleep drive, balanced diets optimize the serotonin–melatonin axis critical for circadian rhythm regulation, and smoking cessation eliminates nicotine’s disruptive stimulation of acetylcholine receptors, collectively reducing sleep fragmentation and stabilizing sleep–wake cycles (59–61). Therefore, LE8 establishes a dynamic interaction network between sleep and cardiovascular health through integrated pathways encompassing direct sleep assessment, metabolic-inflammatory regulation, and behavioral interventions. The DI-GM may improve LE8 through the following pathways. By reflecting diet-induced modulation of microbial diversity, DIGM potentially regulates the production of gut-derived metabolites such as SCFAs and tryptophan derivatives (e.g., serotonin and melatonin precursors) (62). These metabolites act via the MGBA axis to directly enhance sleep metrics of LE8, including prolonged deep sleep duration and reduced sleep onset latency, through central nervous system modulation (62). For instance, SCFAs can cross the blood–brain barrier to normalize HPA axis activity, thereby lowering stress hormone levels and improving sleep quality (63). Furthermore, DIGM-optimized dietary patterns (rich in fiber and polyphenols) not only foster microbial equilibrium but also indirectly boost LE8 scores through systemic anti-inflammatory effects, circadian rhythm regulation, and enhanced intestinal barrier integrity (45, 64, 65).

The mediating role of LE8 as an intermediate variable may arise from the following mechanisms. Dietary components (e.g., tryptophan) serve dual roles as both scoring elements in DIGM and precursors for melatonin synthesis, directly influencing sleep metrics of LE8. DIGM-optimized gut microbiota may transmit signals via vagal pathways with the nucleus tractus solitarius (NTS) in the brainstem, modulating autonomic balance metrics of LE8—such as heart rate variability (HRV)—thereby affecting sleep architecture (64). Conversely, improvements in sleep parameters of LE8 (e.g., extended sleep duration) may reciprocally enhance the abundance of specific microbial taxa (e.g., *Lactobacillus* genus), establishing a positive feedback loop (66). Additionally, UK Biobank research suggests that the DIGM–sleep association may be modulated by polygenic risk scores (PRS). For instance, individuals carrying specific microbiota-related genetic variants exhibit more pronounced improvements in LE8 sleep scores through DIGM interventions (67, 68). Conversely, the non-significant mediation effects of individual mLE8 components underscore the importance of integrated health management.

Our study holds significant clinical implications to demonstrate positive connections between the DI-GM, DI-GM beneficial, and sleep disorders using large-scale population data. The findings suggested that those with DI-GM scores of 5 or above showed a 34% or greater reduction in sleep disorders compared to those scoring 3 or below, while a DI-GM beneficial score ≥6 corresponded to a 35% reduction compared to those with scores of 1 or less. Although causal relationships cannot be definitively established, these findings imply that optimizing gut microbiota composition through dietary modifications may be utilized as a novel method of intervention for preventing or alleviating sleep disorders, such as increasing intake of beneficial ingredients (e.g., cranberries, avocados, broccoli, whole grains, and fermented dairy products) and reducing unfavorable components (e.g., red meat and processed foods). Importantly, beneficial DI-GM components, such as polyphenols in coffee and green tea, may improve sleep quality via mechanisms involving gut–brain axis modulation and systemic inflammation reduction (69). Interestingly, no statistically significant link was found between unfavorable dietary components (DI-GM unfavorable) and sleep disorders, highlighting that prioritizing beneficial food intake over restrictive dietary approaches may be more effective for sleep health. Subgroup analyses demonstrated that this association remained statistically significant among males, current smokers, alcohol consumers, and individuals without hypertension, diabetes, or depression. These findings contribute to the understanding that cross-system regulation of the “diet–microbiota–sleep” axis and advocate for integrating DI-GM assessments into clinical nutrition protocols for sleep disorders patients, offering quantifiable criteria for personalized dietary guidelines. Furthermore, as a composite metric encompassing diet, physical activity, metabolism, cardiovascular health, and mental well-being, even after adjusting for sleep in mediation analyses, mLE8 still mediated the association between DI-GM and sleep disorders.

Nevertheless, our study acknowledges several limitations. First, the cross-sectional structure of NHANES data precludes causal inference, as the temporal sequence between DI-GM and sleep disorders risk cannot be established. Second, sleep disorders were assessed via self-report, introducing potential recall bias and lacking objective validation (e.g., polysomnography). This may lead to underdiagnosis of some patients with sleep disorders, reflecting a limitation of NHANES. Therefore, we included sleep duration as a covariate in our study to minimize measurement bias. Third, although multiple confounders were adjusted for, residual confounding from unmeasured genetic or environmental factors may persist. While subgroup analyses revealed significant associations in smokers and alcohol consumers, heterogeneity across other subgroups (e.g., racial/ethnic groups or individuals with metabolic comorbidities) requires validation in larger samples. Moreover, DI-GM differs from existing indices by incorporating specific food items (e.g., fermented dairy) rather than broad food groups (22, 70), and excludes foods not yet linked to gut microbiota in prior studies (22). The independent mechanisms of specific DI-GM components (e.g., fermented dairy or whole grains) remain unclear, necessitating experimental research to elucidate microbiota-mediated biological pathways. Finally, the use of mLE8 in mediation analyses may not fully capture the original LE8 construct due to the exclusion of sleep components. Future longitudinal cohorts and dietary intervention trials are warranted to confirm the clinical utility of DI-GM-guided strategies for sleep health.

## 5 Conclusion

DI-GM and DI-GM beneficial were both significantly and inversely associated with sleep disorders. Those with DI-GM scores of 5 or above showed a 34% or greater reduction in sleep disorders compared to those scoring 3 or below. Similarly, people with DI-GM beneficial scores of 6 or higher had a 35% reduction than those with scores of 1 or less in sleep disorders. Additionally, mediation analysis revealed that mLE8 played a mediating role in this relationship. Developing integrated intervention programs that combine nutritional counseling and lifestyle management may represent a more optimal approach for patients with sleep disorders.

## Data Availability

The original contributions presented in the study are included in the article/Supplementary material, further inquiries can be directed to the corresponding author.

## Footnotes

^1^ www.medsta.cn/software
