# Supplementary material for: Modified life’s essential 8 mediate the correlation between dietary index for gut microbiota and sleep disorders
Source: Front Nutr. 2025 Jun 25;12:1611714. doi: 10.3389/fnut.2025.1611714 (PMC12237917; doi:10.3389/fnut.2025.1611714)
Supplement: Supplementary file 1 [file Data_Sheet_1.docx]

Supplementary Material

# Supplementary Figures and Tables

## Supplementary Figures


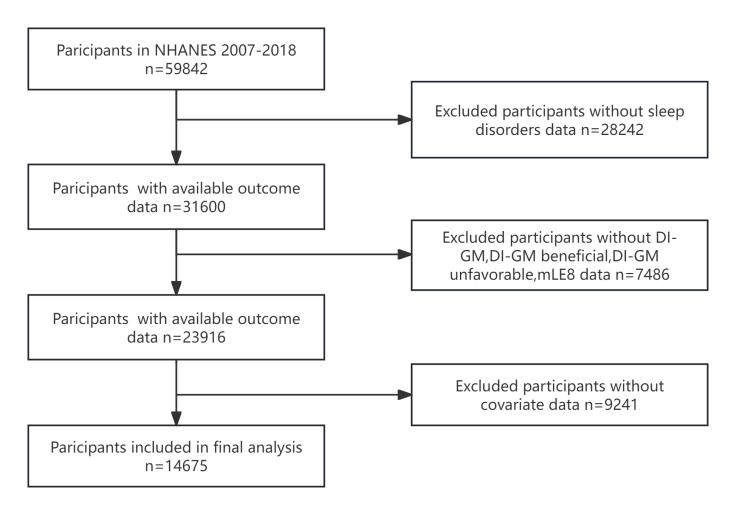


Figure 1. Study flow chart. Abbreviations: NHANES: National Health and Nutrition Examination Survey. DI-GM, dietary index for gut microbiota; mLE8: Modified Life's Essential 8.

**
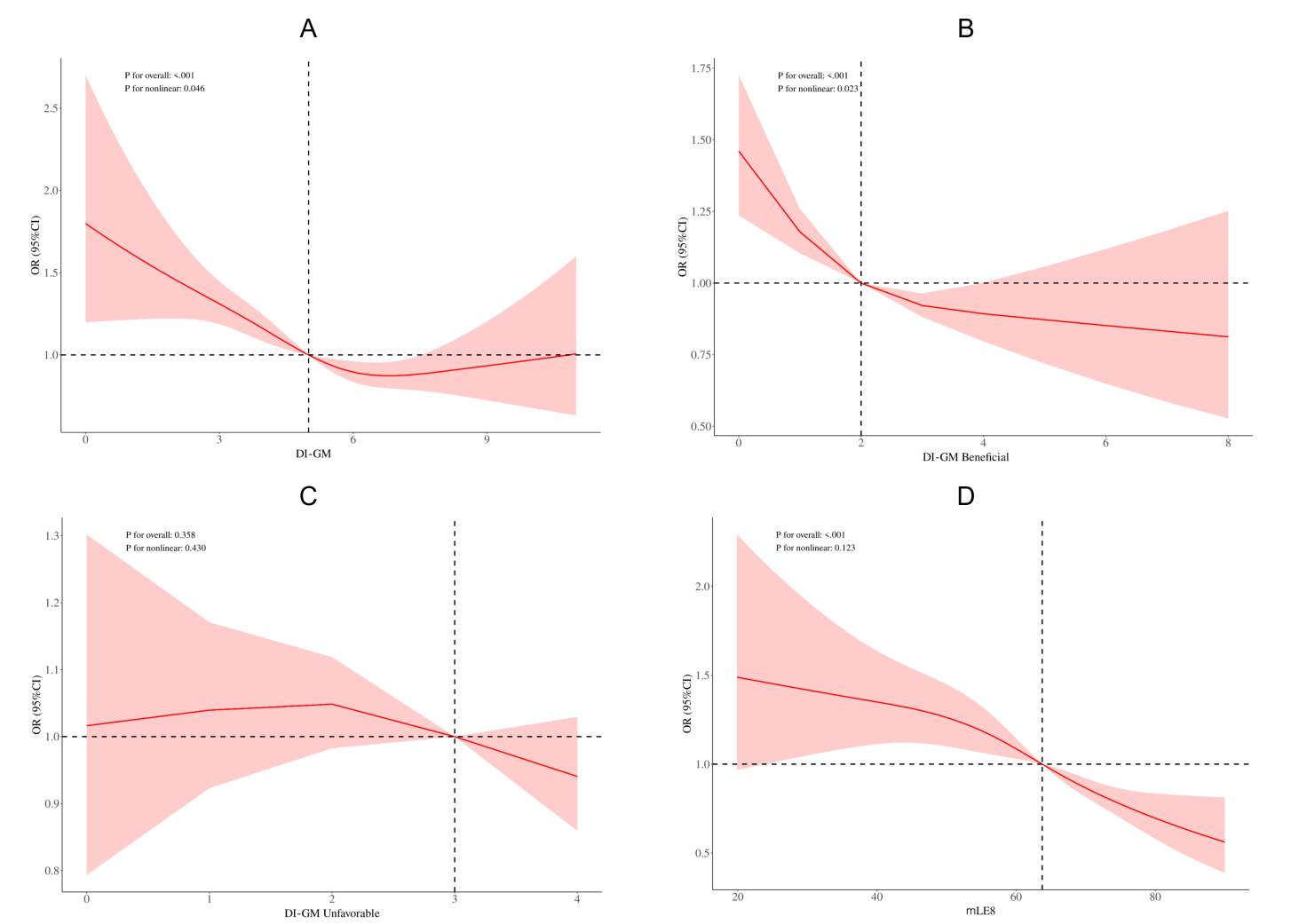
**Figure 2. Dose-response connections of DI-GM, DI-GM beneficial, DI-GM unfavorable and mLE8 with sleep disorders. (A) Connections between DI-GM score and sleep disorders. (B) Connections between DI-GM beneficial score and sleep disorders. (C) Connections between DI-GM unfavorable score and sleep disorders. (D) Connections between mLE8 score and sleep disorders. The model was controlled for variables such as poverty-to-income ratio (PIR), gender, race, age, marital status, educational attainment, and smoking status, drank at least 12 alcoholic drinks in the previous year, BMI, MET categorization, sleep duration, hypertension, diabetes, depression, stroke. Abbreviations: BMI, body mass index; MET, metabolic equivalent of task; PIR, poverty income ratio; mLE8: Modified Life's Essential 8; DI-GM, dietary index for gut microbiota; OR, odds ratio; CI, confidence interval; RCS, restricted cubic spline.


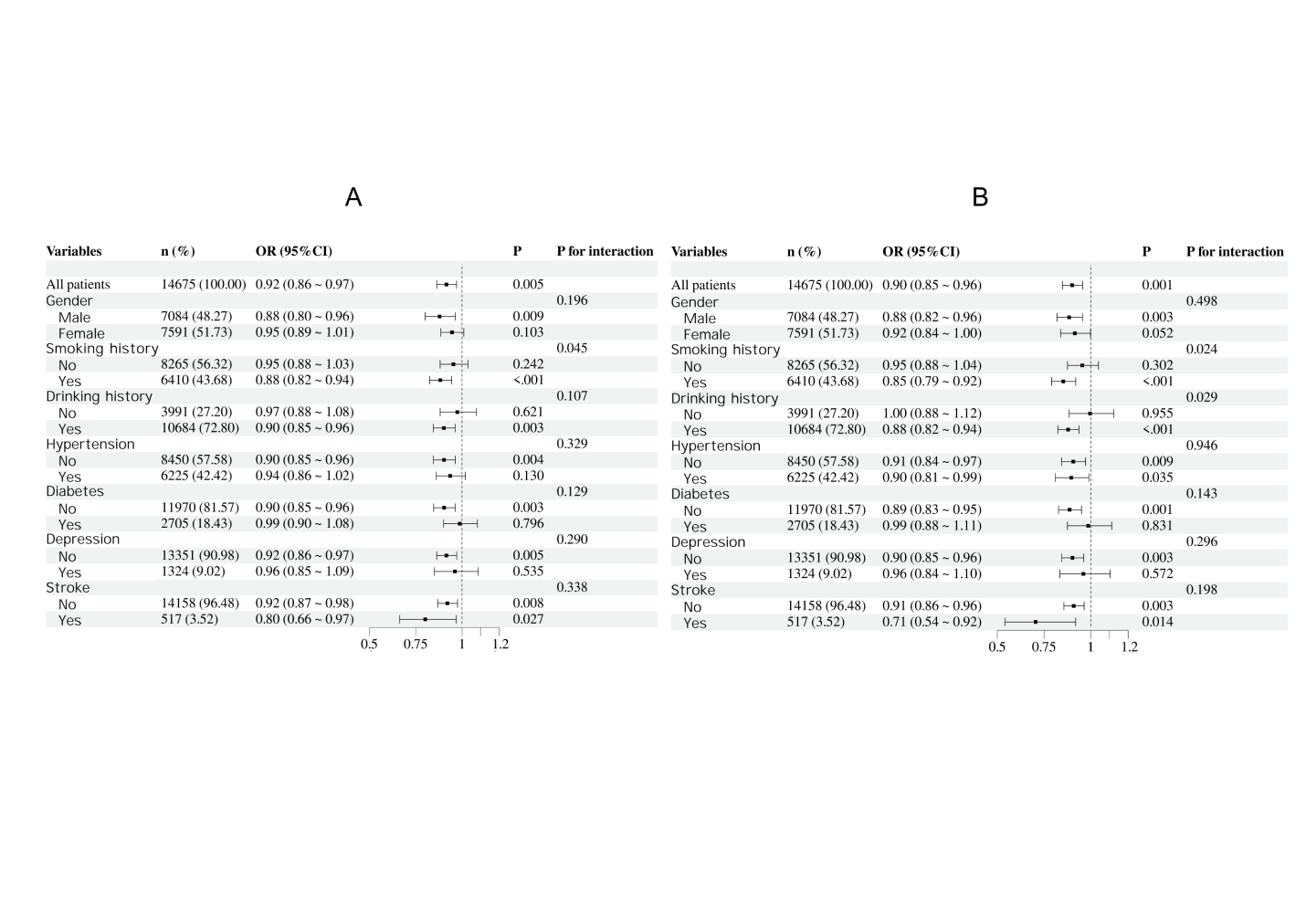


Figure 3. Sensitivity analysis between DI-GM, DI-GM beneficial, and sleep disorders. (A) Subgroup analysis between DI-GM and sleep disorders. (B) Subgroup analysis between DI-GM beneficial and sleep disorders. Analyses were adjusted for gender, age, education level, race, marital status, PIR, smoking status, drank at least 12 alcoholic drinks in the previous year, BMI, MET categorization, sleep duration, hypertension, diabetes, depression and stroke. Alcohol use: drank at least 12 alcoholic drinks in the previous year. Abbreviations: BMI, body mass index; PIR, poverty income ratio; MET, metabolic equivalent of task; DI-GM, dietary index for gut microbiota; mLE8, Modified Life's Essential 8; OR, odds ratio; CI, confidence interval.


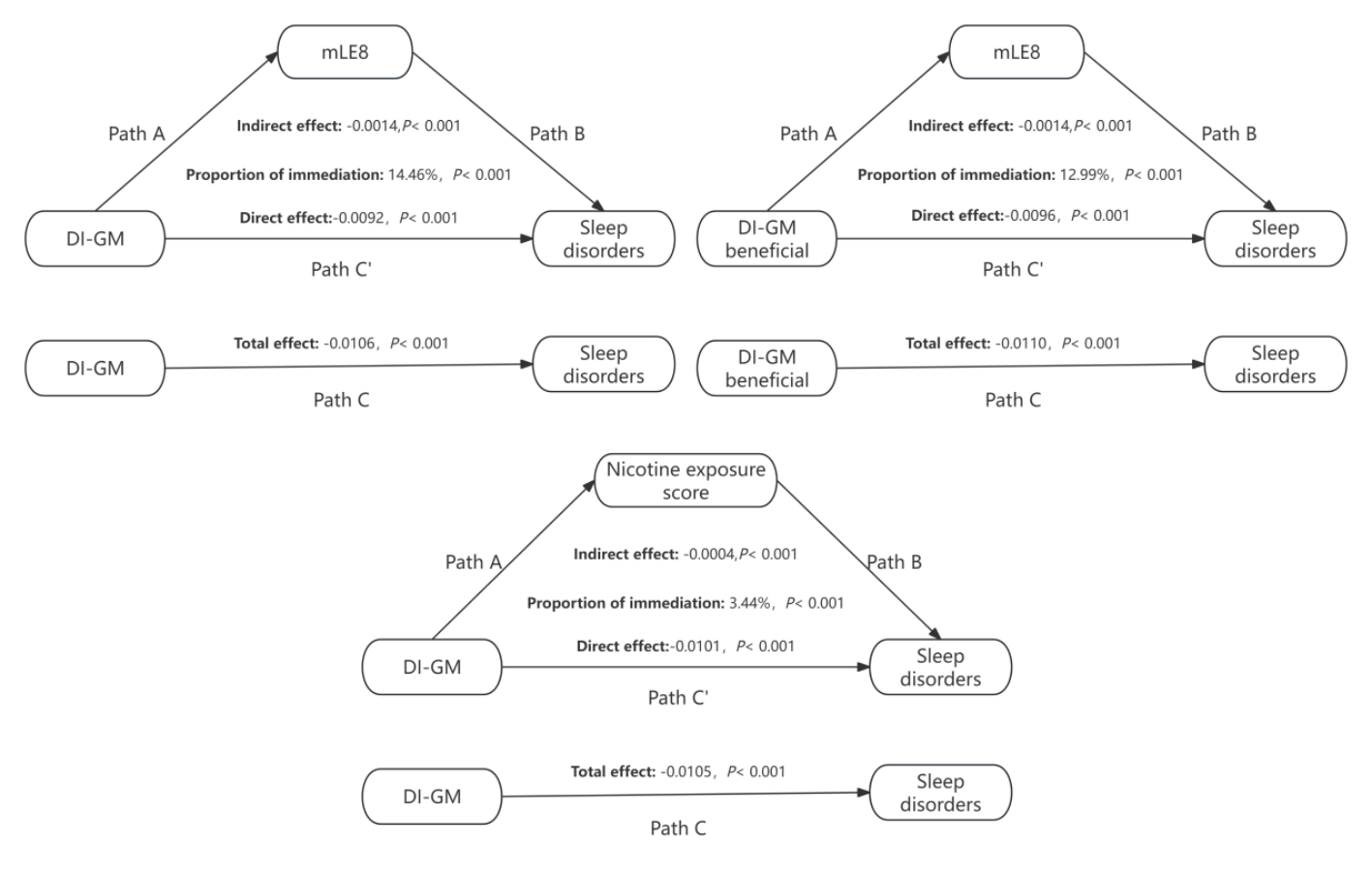


Figure 4. Diagrammatic representation of the mediation effect analysis between DI-GM, DI-GM beneficial, mLE8,nicotine exposure score and sleep disorders. Path C represents the total effect, while path C′ signifies the direct effect. By multiplying paths A and B, the indirect effect is obtained (A×B). The proportion mediated is calculated by taking the indirect effect, dividing it by the combined total of the indirect and direct effects, and multiplying the result by 100%. DI-GM, Dietary Index for Gut Microbiota; mLE8: Modified Life's Essential 8. Adjustments were made to the analyses for: gender, age, PIR, race, marital status, education level, smoking status, drank at least 12 alcoholic drinks in the previous year, BMI, MET categorization, hypertension, diabetes, depression and stroke.

## Supplementary Tables

Supplementary Table 1. Definition of components in DI-GM.

| **Component** | **Included Foods within the Component** | **Scoring** |
| --- | --- | --- |
| Beneficial to gut microbiota |  |  |
| Avocados | Avocados | For each component, a score of 1 if consumption at or above the sex-specific median, else 0 |
| Broccoli | Broccoli |  |
| Chickpea | Chickpeas |  |
| Coffee | Coffee |  |
| Cranberries | Cranberries |  |
| Fermented dairy | Yogurt, cheese, kefir, sour cream, buttermilk |  |
| Fiber | Not applicable |  |
| Green Tea | Green Tea |  |
| Soybean | Soy products—Soy milk, Tofu |  |
| Whole grains | Grains defined as whole grains, containing the entire grain kernel―the bran, germ, and endosperm |  |
| Unfavorable to gut microbiota |  |  |
| High-fat diet (% energy) | Not applicable | 0 if consumption at or above 40% energy from fat, else 1 For each remaining component, a score of 0 if consumption at or above the sex-specific median, else 1 |
| Processed meat | Frankfurters, sausages, corned beef, and luncheon meat that are made from beef, pork, or poultry |  |
| Red meat | Beef, veal, pork, lamb, and game meat; excludes organ meat and cured meat |  |
| Refined grains | Refined grains that do not contain all of the components of the entire grain kernel |  |

DI-GM Scoring Method: Using dietary data from the NHANES database, the sex-specific median intake for each component listed in the table above was first calculated. Then, a score of 1 was assigned to participants whose consumption of each beneficial component exceeded the sex-specific median, and to those whose consumption of each unfavorable component was below the sex-specific median. A score of 0 was assigned to participants whose consumption of each beneficial component was below the sex-specific median, and to those whose consumption of each unfavorable component exceeded the sex-specific median.

Supplementary Table 2. Definition and scoring approach for the American Heart Association's Life's Essential 8 score.

| **Domain** | **CVH Metric** | **Measurement** | **Quantification and Scoring of CVH Metric** | | |
| --- | --- | --- | --- | --- | --- |
| Health  Behaviors | Diet | Healthy Eating Index-2015 diet score percentile | Quantiles of DASH-style diet adherence or HEI-2015 (population)  Scoring (Population):  Points Quantile  100 ≥ 95th percentile (top/ideal diet)  80 75th- 94th percentile  50 50th -74th percentile  25 25th -49th percentile  0 1st-24th percentile (bottom/least ideal quartile) | | |
|  | Physical activity | Self-reported minutes of  moderate or vigorous physical activity per week | Metric: Minutes of moderate (or greater) intensity activity per week  Scoring:  Points Minutes | | |
|  |  |  | 100  90  80  60  40  20  0 0 | | ≥ 150  120 - 149  90 - 119  60 - 89  30 - 59  1 - 29 |
|  | Nicotine exposure | Self-reported use of cigarettes or inhaled nicotine- delivery system | Metric: Combustible tobacco use and/or inhaled NDS use; or secondhand smoke exposure  Scoring:  Points Status  100 Never smoker  75 Former smoker, quit ≥ 5 y  50 Former smoker, quit 1 - < 5 y  25 Former smoker, quit < 1 y, or currently using inhaled  NDS  0 Current smoker  Subtract 20 points (unless score is 0) for living with active indoor smoker in home | | |
|  | Sleep health | Self-reported average hours of sleep per night | Metric: Average hours of sleep per night Scoring:  Points Level  100 7 - < 9  90 9 - < 10  70 6 - < 7  40 5 - < 6 or ≥ 10 | | |
|  |  |  | 20 4 - < 5  0 < 4 | | |
| Health Factors | Body mass index | Body weight (kg) divided by  2 height squared (m ) | Metric: Body mass index (kg/m2) Scoring:  Points Level 100 < 25  70 25.0 - 29.9  30 30.0 - 34.9  15 35.0 - 39.9  0 ≥ 40.0 | | |
|  | Blood lipids | Plasma total and HDL-  cholesterol with calculation of non-HDL-cholesterol | Metric: Non-HDL-cholesterol (mg/dL) Scoring:  Points Level | | |
|  |  |  | 100  60  40  20  0 | <130  130 - 159 160 - 189 190 - 219 ≥ 220 | |
|  |  |  | If drug-treated level, subtract 20 points | | |
|  | Blood glucose | Fasting blood glucose or casual hemoglobin A1c | Metric: Fasting blood glucose (mg/dL) or Hemoglobin A1c (%) Scoring:  Points Level  100 No history of diabetes and FBG < 100 (or HbA1c < 5.7)  60 No diabetes and FBG 100 - 125 (or HbA1c 5.7 - 6.4) (Pre-diabetes)  40 Diabetes with HbA1c < 7.0  30 Diabetes with HbA1c 7.0 - 7.9  20 Diabetes with HbA1c 8.0 - 8.9  10 Diabetes with Hb A1c 9.0 - 9.9  0 Diabetes with HbA1c ≥ 10.0 | | |
|  | Blood pressure | Appropriately measured  systolic and diastolic blood pressure | Metric: Systolic and diastolic blood pressure (mm Hg) Scoring:  Points Level  100 < 120 / < 80 (Optimal)  75 120 - 129 / < 80 (Elevated)  50 130 - 139 / 80 - 89 (Stage IHTN)  25 140 - 159 / 90 - 99  0 ≥ 160 / ≥ 100  Subtract 20 points if treated level | | |

CVH, cardiovascular health; FBG, Fasting blood glucose; HbA1c, Hemoglobin A1c

Supplementary Table 3. Healthy Eating Index-2015 Components & Scoring Standards*.

| **Component** | **Maximum points** | **Standard for maximum score** | **Standard for minimum score of zero** |
| --- | --- | --- | --- |
| Adequacy† | | | |
| Total Fruits | 5 | ≥0.8 cup equiv. per 1,000 kcal | No Fruit |
| Whole Fruits | 5 | ≥0.4 cup equiv. per 1,000 kcal | No Whole Fruit |
| Total Vegetables | 5 | ≥1.1 cup equiv. per 1,000 kcal | No Vegetables |
| Greens and Beans | 5 | ≥0.2 cup equiv. per 1,000 kcal | No Dark Green Vegetables or Legumes |
| Whole Grains | 10 | ≥1.5 oz equiv. per 1,000 kcal | No Whole Grains |
| Dairy | 10 | ≥1.3 cup equiv. per 1,000 kcal | No Dairy |
| Total Protein Foods | 5 | ≥2.5 oz equiv. per 1,000 kcal | No Protein Foods |
| Seafood and Plant Proteins | 5 | ≥0.8 oz equiv. per 1,000 kcal | No Seafood or Plant Proteins |
| Fatty Acids‡ | 10 | (PUFAs + MUFAs)/SFAs ≥2.5 | (PUFAs + MUFAs)/SFAs ≤1.2 |
| Moderation§ | | | |
| Refined Grains | 10 | ≤1.8 oz equiv. per 1,000 kcal | ≥4.3 oz equiv. per 1,000 kcal |
| Sodium | 10 | ≤1.1 gram per 1,000 kcal | ≥2.0 grams per 1,000 kcal |
| Added Sugars | 10 | ≤6.5% of energy | ≥26% of energy |
| Saturated Fats | 10 | ≤8% of energy | ≥16% of energy |

Intakes between the minimum and maximum standards are scored proportionately.

*

†Adequacy components represent the food groups, subgroups, and dietary elements that are encouraged. For these components, higher scores reflect higher intakes, because higher intakes are desirable.

‡Ratio of poly- and monounsaturated fatty acids (PUFAs and MUFAs) to saturated fatty acids (SFAs).

§Moderation components represent the food groups and dietary elements for which there are recommended limits to consumption. For moderation components, higher scores reflect lower intakes, because lower intakes are more desirable.
